# Reasons for parental withdrawal of care in a pediatric intensive care unit in China

**DOI:** 10.1371/journal.pone.0199419

**Published:** 2018-07-25

**Authors:** Kiti Ho, Xia Wang, Lei Chen

**Affiliations:** 1 Department of Pediatrics, Yale University School of Medicine, New Haven, Connecticut, United States of America; 2 Department of Pediatrics, Xiangya School of Medicine, Central South University, Changsha, Hunan, China; Centre Hospitalier Universitaire Vaudois, FRANCE

## Abstract

**Background:**

The past decade saw the establishment of pediatric intensive care units (PICU) across China. This occurred in the context of increasing private shares of medical costs. Payment schemes have not kept pace with the increased availability and demand. As a result a substantial number of parents, in the face of financial constraints, choose to withdraw the medical care of children even when recovery is expected.

**Objective:**

We set out to describe the experience of one PICU in Changsha, an industrialized city near the center of the country with a population of 7.3 million.

**Results:**

During the two-year period 883 patients were admitted to the PICU. One hundred one (11%) patients died during their hospital stay. Of these 69 (68%) died after parents elected to withdraw care. A large proportion (33 out of 69 48%) cited economic factors as a reason behind the decision. Compared with the non-withdrawal group the cases had lower disease severity at admission and on the day of death. On the day of death 34% in the withdrawal group had lower disease severity than at admission, showing clinical improvement. The mean hospital charge for the ICU stay was RMB35,000 (~$5600).

**Conclusion:**

A substantial proportion of patients in a Chinese urban PICU died after parents chose to withdraw their care in the face of financial hardship, even while some were showing clinical improvement. The society has an obligation, and, likely, an economic incentive, to share this burden.

## Introduction

The past decade saw great improvement in the availability of critical care in China. These advances include the establishment of intensive care units, including pediatric intensive care units (PICU), across the vast nation[1]. This development is in the context of a rapidly growing economy. Chinese economy has grown substantially since the liberation of the market place in the 1980’s. Between 2007–2012, the gross domestic product increased by 10.2% annually [2]. Concurrently medical services have expanded. Payment schemes for medical services have also been evolving. Out-of pocket expenses have increased relatively and absolutely. Current challenges include inability to access health care, distrust of the medical professionals, suspicions about efficacy and authenticity of pharmaceuticals, high out-of-pocket medical expenses, and increasing disparities among different regions and different socioeconomic status[3–5].

Health care reforms commenced over the past decade with the goals of expanding health care insurance and decreasing out-of-pocket medical costs. Despite this, the average health expenditure per household is increasing. As a result a substantial number of parents, in the face of financial hardships, choose to abandon the care of children even when recovery, even full recovery, is possible. We set out to describe the experience of one PICU in the large city of Changsha, an industrialized city in the center of the country with a population of 7.3 million.

## Methods

Xiangya hospital PICU is a tertiary care unit with a referral base covering the entire Hunan province (population 65million). There are 10 beds with annual admissions of ~500.

We conducted a retrospective record review of all deaths in the PICU at Xiangya Hospital in Changsha from January 2013 to December 2014. This period represents the beginning of a research collaborative between the US and Chinese Universities. We recorded patients’ demographic information (age, gender, rural/urban) and pediatric critical illness scores (PCIS). We also recorded parental choice to withdraw care and their rationale. Rationales for withdrawal decisions were recorded in the medical record based on unscripted discussions between the clinicians and families We divided children who died in the PICU into “withdrawal group” and “non withdrawal group” based on whether parents chose to withdraw care as documented in the medical record. We compared disease severities at admission and death using PCIS.

Continuous data was reported as means and medians. The PCIS between the withdrawal group and the non-withdrawal group were compared using t-test. Proportions were compared using chi-square or Fisher-Exact tests. Statistical analysis was performed using SPSS 20 (IBM).

PCIS is a scale developed and widely used in China [6]. It is designed to evaluate disease severity in the emergency rooms, PICU, and in-patient settings. PCIS consists of 10 objective measures: heart rate, blood pressure, respiratory rate, PaO2, pH, serum sodium, serum potassium, creatinine, BUN and hemoglobin. It also includes the descriptive assessment for the gastrointestinal system. Scores are assigned for each item. Total maximum score is 100. Score above 80 means disease process is not severe, 71–80 indicates severe disease, and 70 and below means very severe (Table 1)[6].

**Table 1:** PCIS criteria and scoring system [6].

Items	Threshold		Score
	Less than 1 year old:	Older than 1 year old:	
Heart rate (beats/min)	<80 or >180	<60 or >160	4
	80–100 or 160–180	60–80 or 140–160	6
	Other	Other	10
Systolic blood pressure (mmHg)	<55 or >130	<65 or >150	4
	55–65 or 100–130	65–75 or 130–150	6
	Other	other	10
Respiratory rate (breaths/min)	<20 or >70 or obvious irregular breathing pattern	<15 or >60 or obvious irregular breathing pattern	4
	20–25 or 40–70	15–20 or 35–60	6
	Other	Other	10
PaO2 (mmHg)	<50		4
	50–70		6
	Other		10
pH	<7.25 or >7.55		4
	7.25–7.30 or 7.50–7.55		6
	Other		10
Sodium level (mmol/L)	<120 or >160		4
	120–130 or 150–160		6
	Other		10
Potassium level (mmol/L)	<3.0 or >6.5		4
	3.0–3.5 or 5.5–6.5		6
	Other		10
Creatinine (umol/L)	>159		4
	106–159		6
	Other		10
BUN (mmol/L)	>14.3		4
	7.1–14.3		6
	Other		10
Hemoglobin (g/L)	<60		4
	60–90		6
	Other		10
GI system	Bleeding Stress ulcer and ileus		4
	Bleeding stress ulcer		6
	Other		10

This record review was approved by the institutional review board at Central South University (Hunan, Changsha, China) according to the local regulations. It was approved with waiver for informed consent. The data was anonymized prior to the researchers having access to it.

## Results

During the two-year period 883 patients were admitted to the PICU. One hundred one (11%) patients died during their hospital stay. Of these 69 (68%) died after parents elected to withdraw care. Table 2 lists and compares the characteristics between the two groups. There were more patients from the rural area in the withdrawal group compared to the non-withdrawal group (78% vs. 47%). There were no significant statistical differences in the lengths of hospitalization or hospital costs between the two groups.

**Table 2:** Demographics and PCIS.

	Withdrawal group n = 69	Non-withdrawal group n = 32	p-value
Age (years)			
Mean	2.8 (0.08–13)	3.8 (0.17–12)	0.18
Median	1.3	1.6	
Gender			
Male	35 (51%)	18 (56%)	0.29
Female	33 (48%)	14 (44%)	
XXY	1 (1%)	0	
Residence			
Rural	54 (78%)	15 (47%)	< 0.01
Urban	15 (22%)	17 (53%)	
Hospital cost (RMB)	31,215 (2,034–159,015)	45,077 (1,958–222,040)	0.15
Length of stay (days)			
Mean	8.3 (1–41)	9.3 (1–37)	0.42
Median	5	4.5	
PCIS at admission	85.8(68–100)	80.1(60–100)	<0.01
PCIS on day of death	78.7(48–96)	63.9(50–76)	<0.01
Patients with improving PCIS	23/69 (34%)	0/32 (0%)	<0.01

The rationales for the withdrawal decisions are listed in Table 3. In some cases more than one reason was given. A large proportion (33/69 48%) cited economic factors as a reason behind the decision.

**Table 3:** Reasons to withdraw care in the withdrawal group. More than one reasons could be found in patients’ charts.

Withdrawal Reason	N
Financial difficulties	33
Afraid of severe sequelae	15
Poor prognosis as perceived by the family	26
Loss of confidence in the physicians	5
Desire to transfer back to local hospital	2
Total	126

**Fig 1:**
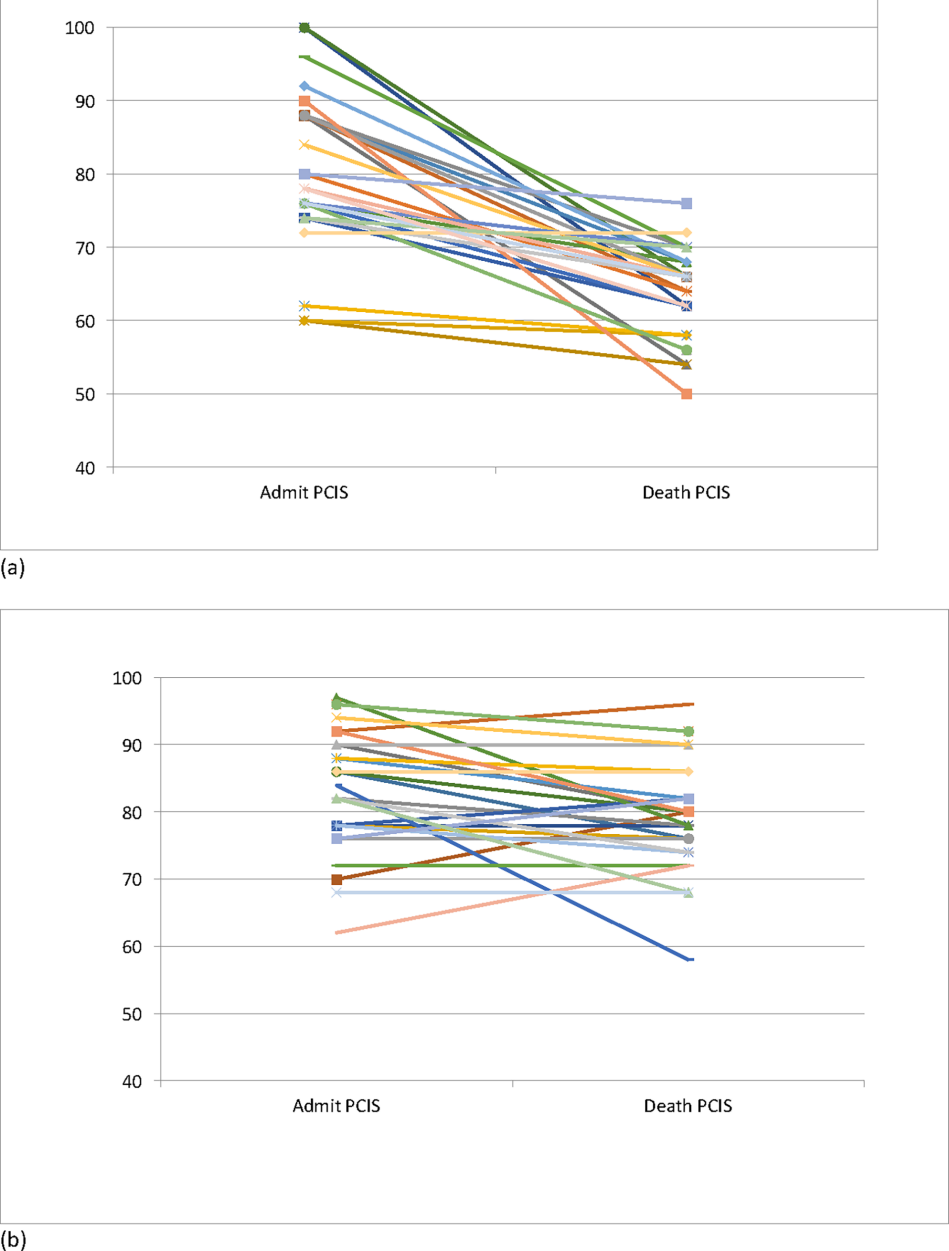
PCIS at admission and death of children. Each line represents one patient. Increasing number implies clinical improvement.

The PCIS at admission and death are listed in Table 4. Compared with the non-withdrawal group the withdrawal group had lower disease severity at admission and on the day of death based on PCIS (Table 4) The decline in clinical status based on PCIS was less in the withdrawal group. On the day of death 23 out of 69 (34%) in the withdrawal group had lower disease severity than at admission, while none in the non-withdrawal group did (Fig 1).

**Table 4:** Mean PCIS on admission and on the day of death.

Average PCIS	Withdrawal group	Non-withdrawal group	p-value
Admission	85.8 (68–100)	80.1 (60–100)	< 0.01
Death/discharge	78.7 (48–96)	63.9 (50–76)	< 0.01
Difference	- 7.1	- 16.2	< 0.01

## Discussion

In this study we demonstrated that a significant number of parents elected to withdraw the care of their children in an urban PICU due to economic burdens. While the current study focused on a PICU in one large tertiary care hospital, this is in the context of larger, evolving social and economic changes in China.

Rapid economic development over the past 3 decades made advanced health care more widely available [1]. Payment schemes for inpatient care have not kept up with the increasing costs. Prior to 2003, there was one public insurance scheme, the Urban Employee Basic Medical Insurance (UEBMI) [6]. Residents in the rural areas were not covered unless they paid for commercial insurance, which many could not afford. In 2003, the government initiated a new insurance scheme called New Rural Cooperative Medical Scheme (NRCMS), under which all rural residents, including children, were eligible. NRCMS is subsidized by central and local government with small contributions from the rural residents [3,4]. By 2011, about 90% of rural residents were covered under the NRCMS [6]. The coverage levels were often low. In 2011, the NRCMS covered only about 50% of the inpatient cost and 60–70% of outpatient cost, despite the increase of reimbursement ceiling per year from $3600 in 2007 to $7692 in 2011 [2].

The situation in the urban population is similar. Urban Resident Basic Medical Insurance (URBMI) was created in 2007 to cover those who were unemployed, had low income, as well as retirees and children.^5^ Similar to NRCMS, the central and local governments provided subsidies to enroll eligible people with amounts varying depending on the geographic location. By 2011, about 90% of the urban population were covered. However, out-of-pocket health expenditure per household increased by 3.7% annually [7]. The urban population faced similar challenges as the rural population, including increased hospital admission rates and insufficient reimbursement. In 2011, the reimbursement rate for inpatient cost was around 55% [7].

These factors contribute to the increasing out-of-pocket health care cost in China. As shown in our study, a significant number of patients are unable to afford the care that they need. Their families make the difficult decisions to forego further medical care even in the face of improving clinical status and, possibly, good prognosis. Similar to our research another recent study showed that 30–40% of children with congenital heart diseases in Zhejiang province were not getting adequate medical care because of high health care cost, despite increased insurance coverage [8].

Several developments may lead to change in the coming years. The Chinese government implemented the 12th Five Year Plan in 2011 with the goal to provide affordable, quality basic health care with equitable access among different socioeconomic groups by 2020 [2]. The government is increasing its financial contribution to NRCMS and URBMI, which increases the coverage percentage. The government will also continue to improve the quality of primary health care by providing training for the general practitioners and expanding the primary care infrastructure. A national essential drug list is under close monitoring to ensure the quality and affordable prices of these important medications. Public hospital reform is also an important area of focus for the 12th Five Year Plan. The government hopes to reform the payment schemes to uncouple drug sale revenue from providers' income (a source of public distrust) and to improve quality of the services. The government also hopes to increase private hospital market share to 20% by 2015, relying on competition from the private sector to promote improvement [2].

Our study has several limitations. We do not have data on the insurance coverage of each patient. We know from literature that on the average the various public insurance schemes cover about half of the inpatient medical costs. The total costs were similar between the two groups even though the disease severity was lower in the withdrawal group. The reasons may be due to shorter hospitalizations in the withdrawal group, although we didn’t evaluate other factors. PCIS does not capture a comprehensive view of the pathophysiology and the prognosis of each patient. The rationales behind withdrawal were recorded by the physicians after conversations with the parents. These conversations did not follow standardized scripts. The reasons are not mutually exclusive nor exhaustive. As a consequence other factors, such as religion, social-economic status, and educational backgrounds that undoubtedly contribute to the difficult decisions to withdrawal were not captured in the database. Future prospective study incorporating these factors would be enlightening.

## Conclusions

A substantial proportion of patients in a Chinese urban PICU died after parents chose to withdraw their care in the face of financial hardship, even while some were showing clinical improvement. The society has an obligation, and, likely, an economic incentive, to share this burden.

## Supporting information

S1 Table Is the primary data file. (XLSX)

## Abbreviations

PICU: Pediatric Intensive Care Unit
RMB: Renminbi, Chinese currency denomination
PCIS: Pediatric Critical Illness Score
